# Hyperosmotic phase separation: Condensates beyond inclusions, granules and organelles

**DOI:** 10.1074/jbc.REV120.010899

**Published:** 2020-11-23

**Authors:** Ameya P. Jalihal, Andreas Schmidt, Guoming Gao, Saffron R. Little, Sethuramasundaram Pitchiaya, Nils G. Walter

**Affiliations:** 1 Single Molecule Analysis Group and Center for RNA Biomedicine, Department of Chemistry, University of Michigan, Ann Arbor, Michigan, USA; 2 Cell and Molecular Biology Graduate Program, University of Michigan, Ann Arbor, Michigan, USA; 3 Biophysics Graduate Program, University of Michigan, Ann Arbor, Michigan, USA; 4 Program in Chemical Biology, University of Michigan, Ann Arbor, Michigan, USA; 5 Michigan Center for Translational Pathology, University of Michigan Medical School, Ann Arbor, Michigan, USA; 6 Department of Pathology, University of Michigan, Ann Arbor, Michigan, USA

**Keywords:** macromolecular crowding, stress response, protein domain, aggregation, biophysics, fluorescence, membraneless organelles, cloud formation, mesoscale organization, ALS, amyotrophic lateral sclerosis, CPSFs, cleavage and polyadenylation factors, GEMS, genetically encoded nanoparticles, HOPS, hyperosmotic phase separation, ISR, integrated stress response, LCST, lower critical saturation temperature, LLPS, liquid–liquid phase separation, MLOs, membraneless organelles, RNP, RNA–protein, SGs, stress granules, UCST, upper critical saturation temperature

## Abstract

Biological liquid–liquid phase separation has gained considerable attention in recent years as a driving force for the assembly of subcellular compartments termed membraneless organelles. The field has made great strides in elucidating the molecular basis of biomolecular phase separation in various disease, stress response, and developmental contexts. Many important biological consequences of such “condensation” are now emerging from *in vivo* studies. Here we review recent work from our group and others showing that many proteins undergo rapid, reversible condensation in the cellular response to ubiquitous environmental fluctuations such as osmotic changes. We discuss molecular crowding as an important driver of condensation in these responses and suggest that a significant fraction of the proteome is poised to undergo phase separation under physiological conditions. In addition, we review methods currently emerging to visualize, quantify, and modulate the dynamics of intracellular condensates in live cells. Finally, we propose a metaphor for rapid phase separation based on cloud formation, reasoning that our familiar experiences with the readily reversible condensation of water droplets help understand the principle of phase separation. Overall, we provide an account of how biological phase separation supports the highly intertwined relationship between the composition and dynamic internal organization of cells, thus facilitating extremely rapid reorganization in response to internal and external fluctuations.

In eukaryotic cells, the densely packed intracellular environment is compartmentalized to allow specific biochemical reaction pathways to be efficiently regulated in a complex, highly heterogeneous environment where individual catalysts and reactants are present at low concentrations. While membrane-bound organelles have been considered paradigmatic of mechanisms that localize biochemical processes, studies from the past decades have brought increased attention to a more adaptive and dynamic strategy for intracellular spatial organization using “membraneless” organelles (MLOs). These amorphous structures are ubiquitous, are observed across cellular compartments and even in the extracellular space, and are characterized by their lack of a lipid boundary. They are heterogeneous in composition and size, typically ranging from 0.01 to 10 μm, and are subjects of active study owing to their propensity to dynamically assemble and disassemble, priming the cell for rapid responses to intrinsic and extrinsic perturbations (1, 2, 3). The prevalence of condensates in all forms of life and the seemingly fundamental rules that govern condensate assembly suggest that these structures and mechanisms may go back to the origins of life itself (4, 5).

Since the early days of microscopy and cell biology, cytologists have reported observations of “lifeless bodies,” “granules,” “inclusions,” and other membraneless structures (6, 7). Despite being observed for over a century, they have come to be extensively studied only in the past decade, largely owing to advances in contemporary technologies that allow probing these structures at unprecedented spatiotemporal resolution, both *in vitro* and *in situ*. In addition to technical innovations, our understanding of these mesoscopic structures has been shaped by the metaphors used to describe MLOs over the years. This review aims to provide an overview of these different terminologies and put them in perspective of recent insights into hyperosmotic phase separation (HOPS) of the multimeric proteome.

## A brief history of intracellular condensation

Membraneless structures such as the nucleolus, nuclear speckles, and some RNA–protein (RNP) granules have been studied since the first half of the 20th century, although the earliest reports of such structures go back to the 1800s (8). The most prominent of these structures, the nucleolus, was first described as an “organelle,” in the sense of a distinct compartment with an associated function (9). Thus, the earliest descriptors to signify subcellular compartmentalization were borrowed from canonical membrane-bound organelles and simply denoted observable subcellular organization. While this view provided a framework to relate the observable structure of such compartments with their biochemical properties and functions, it did not provide a way to understand the physical origins of nucleoli.

The first decade of the 21st century saw attention turning to the function of various, newly discovered classes of membraneless structures. Structures such as P-bodies, stress granules (SGs), purinosomes, and G-bodies were described as “granules,” “compartments,” or “clusters” (5, 10, 11), terms that emphasize the appearance of such structures under the light or fluorescence microscope. These terms marked, however, a departure from “organelles”—they did not necessarily have associations with differentiated biological function (12). This was closely followed by first reports of the dynamic biophysical properties of these structures. Handwerger *et al.* (13) recognized that nuclear condensates, which the authors reported to be “porous” and “sponge-like,” are materially continuous with the nuclear matrix and do not pose a barrier to diffusion, while still being compositionally distinct from the nucleoplasm. Brangwynne *et al.* (14) noted that cytoplasmic RNP “granules are…biophysically similar to the rest of the intracellular fluid, and yet appear to represent a different ‘state’ of cytoplasm, comprised of a locally distinct molecular ensemble”. These observations broadened the inquiry into MLOs to include the study of common principles underlying their origins and revealed several unexpected features, such as liquid-like characteristics, liquid-to-solid transitions, etc. The various contexts in which MLOs are now known to exhibit dynamic fluid properties such as droplet fusion, surface tension, dripping, wetting, and viscoelasticity have been reviewed elsewhere (15, 16, 17, 18).

Since the 2010s, the term “membraneless organelle,” originally used to describe the nucleolus, started to be applied in a more general sense to RNP granules and other “assemblies/assemblages” that show fluid-like properties (14). This broadening of the term from one specific structure to an entire category of structures similarly marked the start of a unification and ascension of the study of MLOs, whose biological functions were previously underappreciated and considered unrelated.

With increasing interest in phase separation as the basis of the formation of MLOs, the introduction of the phrase “biomolecular condensates” in 2017 has helped bridge the gap between physiological *in situ* observations of such structures and inquiry into their biophysical origins. The term “condensate” explicitly refers to the process of MLO formation and, in doing so, goes beyond the signifier of mere organization connoted by “droplet/MLO” to make a firmer claim about a specific mechanism of formation *via* phase transition (19, 20). Converging on a consensus of terminology, the field has seen an increase in efforts to elucidate the macromolecular structural and sequence features that promote MLO assembly *in vivo* and to study the physiological roles of such structures in development, stress response, and disease (21).

Significant attention has been focused on the phase separation processes in pathological contexts. Prominently, toxic protein aggregation such as those formed by β-amyloid peptide (Aβ) and tau proteins in Alzheimer’s disease, TDP-43/FUS in amyotrophic lateral sclerosis (ALS), and huntingtin protein in Huntington’s disease have been studied as archetypical phase separation processes (22, 23, 24, 25, 26, 27). In this review, we aim to provide a unifying account of intracellular phase separation in which widespread condensation across the proteome, representing the basal tendency of the intracellular environment, is co-opted to sense and appropriately respond to environmental fluctuations and can go awry in disease. We take a physically motivated view of the cell in which the interior of the cell is poised on the brink of phase separation (7, 28, 29). To properly understand the implications of this broadly adaptive cellular behavior, we will first review the theory of phase separation and some important contexts in which cells respond to environmental fluctuations by physicochemical condensation.

## Physicochemical underpinnings of phase separation

**Figure 1:**
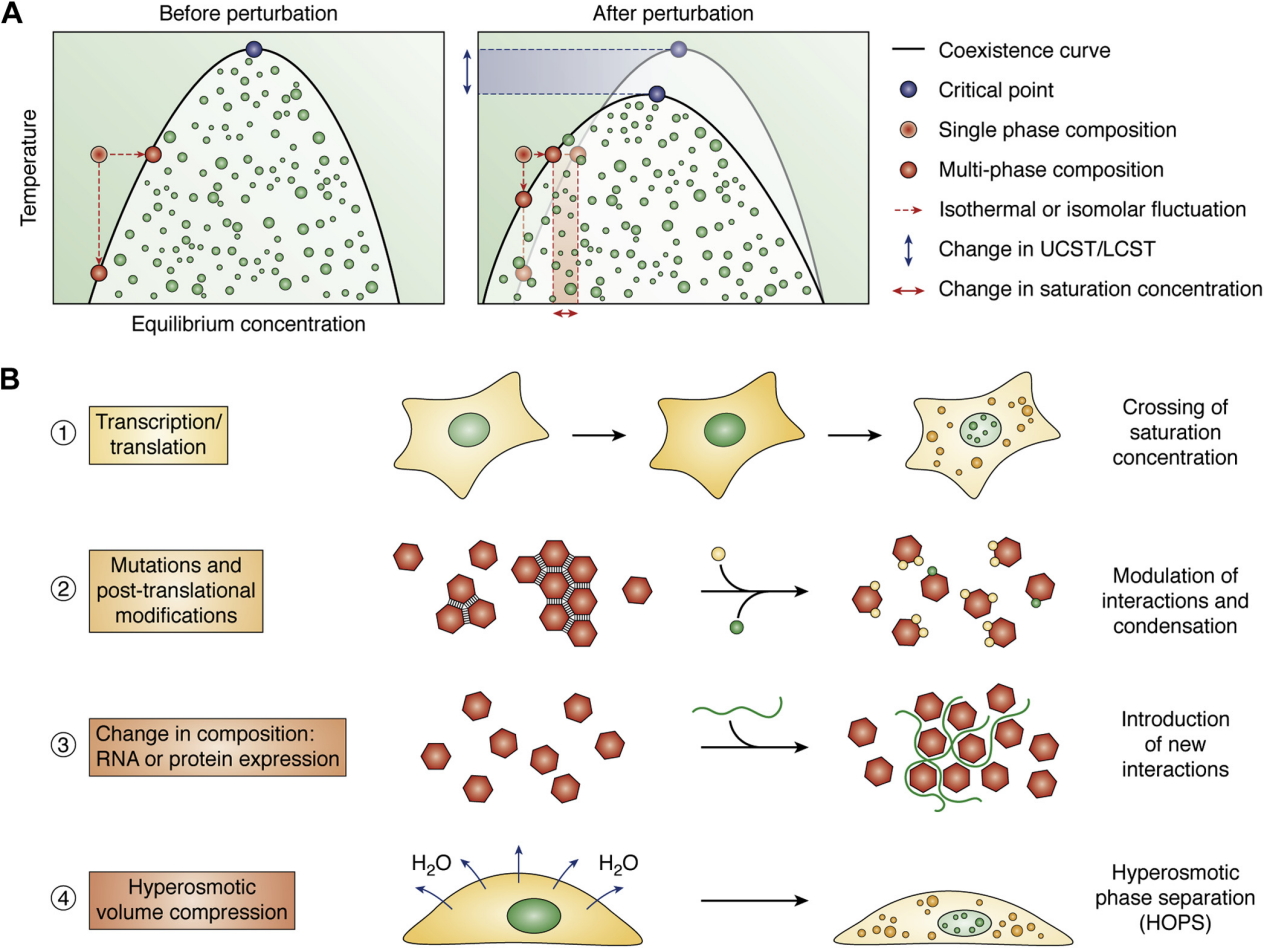
**Phase separation induced by biological perturbations**. *A*, a phase diagram shows the one-phase and multiphase regions in temperature–composition space (left). Changes in temperature and concentration cause the system to transition between the single-phase region and multiphase region, shown as isothermal concentration changes or isomolar temperature changes. Biological perturbation can impact the phase diagram itself, affecting saturation concentrations and upper and/or lower critical saturation temperatures (UCST/LCST, right). *B*, 1. RNA or protein expression changes their concentration until the saturation concentration is crossed. 2. Posttranslational modifications such as methylation and phosphorylation or dephosphorylation alter the association strengths of the solutes and can serve as biological mechanisms to modulate condensation. 3. Changes in intracellular composition by altered expression of RNAs or proteins can modify the phase behavior by introducing new interactions. 4. Hyperosmotic volume compression leads to a sudden jump in concentration and crowding, resulting in hyperosmotic phase separation or HOPS.

Biological liquid–liquid phase separation (LLPS) originates from the weak protein–protein, protein–RNA, and RNA-RNA interactions that drive intracellular solutes to partition out of the dilute phase and preferentially into a condensate, the concentrated dense phase. One important tool to study equilibrium phase separation behavior of a solute is the phase diagram (Fig. 1*A*). A phase diagram is a graphical representation of the thermodynamics of phase separation. It depicts all possible phase states of the system in N-dimensional phase space, where N is the number of external factors that determine the relative contribution of interactions to the free energy of the system (30). Key factors relevant to biological phase transitions include temperature, concentration, valency, and interaction strength. A critical point in this N-dimensional phase space is the threshold beyond which the differences between phases vanish and thus no phase separation is possible, and the system is said to be well mixed. If one factor, say temperature, is fixed at or above its value of the “critical saturation temperature,” phase separation will not occur regardless of the value of all other influencing factors. Biological systems have been observed to show both upper and lower critical saturation temperature (UCST and LCST) behaviors, which determine whether increasing temperature will shift the system out of or into the two-phase region, respectively (31). At any given temperature, the minimal concentration that causes the solute to start undergoing condensation is called the “saturation concentration,” and increasing the concentration further will cause the system to enter the two-phase (“demixed”) region. The effects of isothermal concentration changes and isomolar temperature changes on phase behavior are depicted in Figure 1*A*, left. Biologically relevant perturbations, in addition to changing component concentrations, may end up reshaping the phase diagram itself, only then allowing the system to undergo phase separation at lower concentrations or temperatures (Fig. 1*A*, right).

Extensive efforts have been dedicated to elucidating the molecular features that drive intracellular phase separation (32, 33). The most general requirement is multivalency, which allows molecules to form large assemblies *via* multiple intermolecular contacts. Within protein–protein interfaces, arginine–glycine–glycine/arginine–glycine motifs (34), π–π (35), cation–π, and charge–charge interactions, among others, have been shown to drive protein phase separation (36, 37, 38, 39, 40). These interactions stimulate the higher-order assembly of prion-like domains in protein misfolding diseases (32), together with disordered regions and RNA-scaffolded assembly (41, 42, 43). Additionally, structured protein domains are now emerging as mediators of widespread intracellular phase separation under conditions of high concentration and molecular crowding (44, 45). Altered expression of RNA and protein components therefore can drastically influence both condensation and phase behavior itself (Fig. 1*B*).

Disrupting any of these key interactions driving phase separation is expected to interfere with the phase separation potential of a system. Consistent with this expectation, posttranslational modifications such as phosphorylation and methylation have been found to modulate condensation responses (46, 47, 48, 49) (Fig. 1*B*). The effects of phosphorylation in particular can be dramatic so that, for example, the kinase DYRK3, which prevents condensation of splicing factors in M phase, has been appropriately referred to as a “dissolvase” (46). Similarly, SG assembly in response to various stresses depends on phosphorylation of G3BP and PABP (50). Accordingly, posttranslational modifications are emerging as key modulators of phase separation (48, 51, 52).

A perturbation of biological interest is concentration change arising from altered gene expression or nucleocytoplasmic trafficking, processes commonly associated with developmental changes, signaling, and disease (Fig. 1*B*). While the impact of changing concentration on phase separation by itself is straightforward to study with purified recombinant proteins, there are important caveats to be considered when relating such *in vitro* observations to intracellular concentration changes. Notably, any condition that alters intracellular concentration entails simultaneous changes in multiple factors that influence the phase separation outcome. Hyperosmotic compression, for instance, leads to a decrease in diffusion rates of large macromolecules, an increase in molecular crowding, and possible ionic imbalances in addition to changes in effective concentrations of biomolecules; we elaborate on this multiplicity of changes associated with HOPS later in the text. These effects are similar to changes that have been reported in bacteria, yeasts, and protists in response to glucose starvation, in which volume change causes a fluid-to-glass transition of the intracellular space, simultaneously impacting diffusivity as well as intracellular pH (53, 54, 55). In both these perturbations, the phase separation outcome depends on the compound effects of each of these factors in reshaping the phase boundaries and altering the saturation concentration (Fig. 1, *A*–*B*). Furthermore, such perturbations typically represent dynamic nonequilibrium situations within a complex matrix of competing cellular interaction partners (56), which adds to the complexity of studying intracellular phase separation (57, 58).

**Figure 2:**
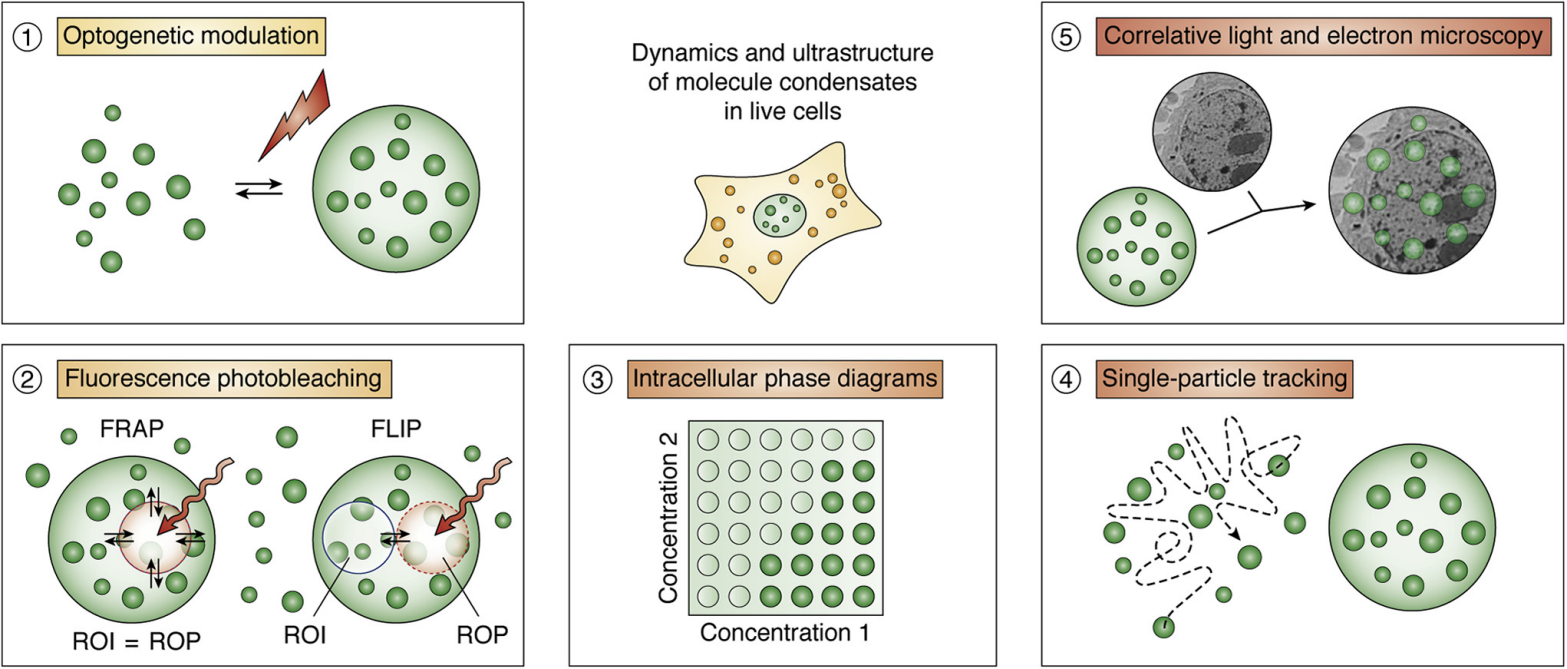
**Observing and quantifying the dynamics and ultrastructure of condensates in live cells.** Counterclockwise from top left: 1. Optogenetic manipulation of condensates dissociates condensation of specific cellular components from cell-wide effects of environmental perturbations. Condensation can be modulated by activating light-sensitive protein-interaction domains using specific wavelengths (126, 127, 128). These methods are powerful as they can modulate phase behavior of specific components without otherwise perturbing the cell; they alter material properties of condensates rapidly, dissect the contributions of individual interactions without being constrained by the cellular milieu; and enable studies of the biochemical impact of rapidly altered molecular clustering in the cell (41, 126, 129, 130). 2. In fluorescence recovery after photobleaching (FRAP), a small region is photobleached and the rate of the recovery of fluorescence in this region serves as a readout of the effect of the local environment on the molecule of interest. For this reason, FRAP has been widely used to assess liquid-like properties (131, 132). ROI, region of interest; ROP, region of photobleaching. Fluorescence loss in photobleaching (FLIP) is similar to FRAP but is used to investigate the exchange of material between condensates. In FLIP, a small region is repeatedly photobleached and the loss of fluorescence is measured in another region (131). 3. Studying phase separation from purified components in the test tube allows precise and systematic investigation of the effects of composition, temperature, pH, salt, etc., to build a phase diagram (133, 134, 135). While such manipulations are less easily achieved in cells, several recent studies have reported strategies to obtain phase transition information from intracellular fluorescence measurements (42, 43, 49, 78). 4. Single particle tracking (SPT) is a powerful tool to study dynamic recruitment of molecules to condensates (45, 65, 136). 5. CLEM, correlative light and electron microscopy is an emerging tool that holds great potential to uncover ultrastructural details of condensates. It involves two steps. In the first step, a fluorescence-tagged moiety is detected to extract spatial information with biomolecular specificity. In the second step, the same sample is then imaged using electron microscopy. Correlating features in the electron micrograph with the fluorescence signal can identify specific components within the ultrastructure of liquid-like condensates, which often is more challenging to achieve *via* electron microscopy alone (137, 138).

Inside cells, condensates show several distinct characteristics and behaviors. Different classes of condensates show physical associations with each other that can be important for seeding condensates. Prominently, P-bodies are thought to seed SGs while sharing components with them (42). Many MLOs have been shown to have ultrastructures, where each condensate often contains a “core” and a “shell” of distinct compositions or subcompartments that differ in material properties (59, 60, 61, 62, 63, 64). This ultrastructural organization of condensates is increasing being studied using superresolution fluorescence methods (63, 65, 66). Recently, single-molecule tools have been applied to study the recruitment of molecules to RNP condensates by tracking RNA localization dynamics (67, 68, 69). These methods, in conjunction with structure determination approaches, are beginning to reveal a complex dependence on RNA conformation and translation while emphasizing the importance of weak/noncanonical RNP interactions in the formation of RNP condensates (66, 70, 71, 72, 73, 74, 75) (Fig. 2). Whereas the quantification and manipulation of condensates in living cells pose challenges, in general fluorescence-based imaging techniques and intracellular modulation assays are proving powerful in studying and quantifying phase separation directly in live cells (Fig. 2).

## Phase separation in response to environmental fluctuations

Eukaryotic cells, from yeast to human, respond to a wide variety of cell-intrinsic and -extrinsic fluctuations by condensation of proteins and RNAs (19). The induction of P-bodies and assembly of SGs are two highly studied, and evolutionarily conserved, stress adaptation mechanisms that are triggered downstream of the integrated stress response (ISR) (50). The ISR is a multistep signaling cascade activated in response to, for example, viral infection, nutrient deprivation, heat shock, oxidative and endoplasmic reticulum stress, and enhances cell survival by altering global protein translation (76). While the downstream pathways of ISR are shared, the sensor of each individual type of stress is distinct, conferring a certain degree of specificity to each stress. For instance, Pab1 (polyA binding protein) and Pub1 (polyU binding protein), two highly expressed proteins in yeast, are differentially enriched within SGs during temperature shock and pH shock, respectively (50, 77). Once the pathways are triggered, the pool of nontranslating mRNA–protein complexes along with phosphorylation of SG component proteins participate in a network of multivalent interactions, ultimately triggering the assembly of SGs (42, 43, 50, 78). In addition to regulating protein translation, cells suspend protein and ribosomal RNA (rRNA) metabolism by sequestering misfolded proteins and nuclear RNA-binding proteins in the nucleolus in response to impaired rRNA processing and DNA damage (79, 80). Proteins that are directed to nucleoli under these conditions are thought to undergo translocation to these sites *via* their interactions with stress-associated noncoding RNAs.

Emerging evidence suggests that condensation responses are also involved in cell signaling cascades that aid cellular homeostasis in response to physiological cues. Condensates at cell membranes (81) and in the cytosol have been shown to regulate cell division, migration and invasion (82, 83), transgenerational memory (84, 85, 86), and immunomodulation (87) in response to a variety of morphogens and endo/para/autocrine signals. In addition to acting across a range of timescales, condensation in response to external perturbations plays a critical role in shaping the spatial organization of cells by moving RNAs and proteins into dynamic MLOs with complex organization, suggesting an intimate relationship between macromolecular sequence, intracellular organization, and the extracellular environment (66, 88, 89, 90).

## Osmotic perturbations and the hyperosmotic phase separation response

We reported in Jalihal, Pitchiaya *et al.* (49) that a significant fraction of the mammalian proteome responds very rapidly, on the order of 10 s, to osmotic cell volume shrinkage by reversibly forming a large number of small “HOPS” condensates. Unlike other constitutively present or stress-induced condensates, which are known to be driven by disordered protein regions, HOPS is predominantly associated with structured homomeric self-interaction domains of proteins, embedded in a significant fraction of the proteome. This rapid response reorganizes both the nucleus and the cytosol. While a majority of previously reported condensation responses occur over a timespan of minutes to hours, it is notable that our observation of HOPS, along with evidence from Cai *et al.*, suggests that cellular response by condensation may also occur much more rapidly, at the timescale of seconds (49, 91). In addition to being ubiquitous and extremely rapid, sustained HOPS influences translation of mRNA targets of microRNAs (69) and impacts cleavage and polyadenylation of nascent transcripts (49), among other gene regulatory processes (92).

Hyperosmolarity exceeding the physiological osmotic range of 285 to 295 mOsm/kg leads to loss of intracellular water through aquaporin channels, manifesting as rapid changes in cell shape and volume across a wide range of tissue types (49, 93, 94). Prolonged exposure to high levels of osmolarity can adversely affect protein structure and lead to DNA damage (95), with long-term exposure leading to cell death by apoptosis and drastic consequences at the organismal level (96). In various cell types, integrins and extracellular matrix components have been implicated in sensing osmotic changes (97). These proteins in turn can activate downstream kinases, leading to activation of specific stress-response genes. The timescale of condensation in HOPS corresponds to early events such as cell shrinkage due to exosmosis, which occurs over 10 s, suggesting that it may occur in parallel to, if not before, the activation of these sensing pathways (49).

As an example, DCP1A, a P-body marker (69), rapidly responds to hyperosmotic shock by undergoing HOPS (49). The degree of DCP1A partitioning into condensates is influenced by both the concentration of the protein and the osmolarity of the medium. Furthermore, DCP1A’s trimerization domain is sufficient to recapitulate this response. The trimerization domain, like other annotated self-interacting domains, is characterized by hydrophobic patches (98). While the involvement of hydrophobic interactions in promoting phase separation has been demonstrated *in vitro* (99, 100), our observation of widespread HOPS of self-interacting proteins of valency ≥2 suggests that hydrophobic interactions in homomultimeric domains may serve as proteome-wide sensors of osmotic change more generally (49).

HOPS is markedly different from the process of SG assembly, which arises from interactions across a core protein–RNA interaction network in response to a rise in nontranslating RNA levels. SG assembly takes significantly longer than HOPS and occurs as a switch-like response to arsenite stress, typically 10 to 30 min after induction of stress (42, 43, 101). This delay presumably reflects the time required to activate the ISR pathway and phosphorylate the appropriate components before they can form condensates. In contrast, DCP1A’s condensation response upon HOPS, like that of several other homomultimeric proteins revealed by a proteomic screen, is a graded response to osmotic compression that is dramatically dependent on the magnitude of the change in osmolarity, showing a 100-fold increase in condensate number upon a twofold increase in osmolarity (49). While phosphorylation modulates the degree of DCP1A condensation in HOPS, it does not dictate the phase separation, suggesting that homomultimeric proteins may sense osmotic fluctuations in their native states, without the need for additional posttranslational modifications.

Proteins that undergo HOPS form largely distinct, nonoverlapping condensates, suggesting that phase separation can dramatically reorganize the intracellular space very rapidly upon osmotic challenge (49). Indeed, such behavior has been previously predicted based on theoretical grounds (29). Such widespread changes in subcellular localization are predicted to have consequences on the associated biochemical pathways. Notably, HOPS-mediated sequestration of the cleavage and polyadenylation factors (CPSFs) away from transcription termination sites provides an elegant explanation for the widespread transcriptional readthrough observed upon osmotic shock that, like HOPS itself, is found to be reversible upon restoring the medium’s tonicity (49). A separate observation suggests that HOPS-like condensation of the YAP protein is associated with changes in YAP-associated gene expression (91). These findings open up new directions of inquiry into the relationship between microscopic cellular organization and phenotype in response to osmotic variation. As such, the discovery of HOPS serves as a starting point to understand how physicochemical and spatial modulation of disparate biochemical pathways may converge on the ultimate goal of shaping the cell’s response under duress.

In addition to HOPS in mammalian cells, evidence is emerging that osmotic stress in other eukaryotes elicits a rapid phase-separation-like response. Notably, a similar response is observed in yeast, suggesting that the sensitivity of proteomes to osmotic changes may be an evolutionarily ancient adaptation and may point to a broader class of mechanisms that use phase separation to sense and rapidly respond to osmotic fluctuations (77, 102).

## Crowding, depletion attraction, and confinement

What is the mechanistic relationship between osmotic volume change and phase separation? Osmotic cell compression changes cell volume by exosmosis, resulting in an increase in intracellular crowding and effective protein concentration, both of which influence phase separation as discussed above (103, 104). Molecular crowding also has significant effects on protein structure and function (105, 106, 107, 108). Finally, crowding previously has been predicted on theoretical grounds to serve as a cell volume sensor, even though a mechanistic basis is only now emerging (96). For an in-depth review of crowding effects and phase separation, we refer the reader to Andre and Srpuijt 2020 (104).

**Figure 3:**
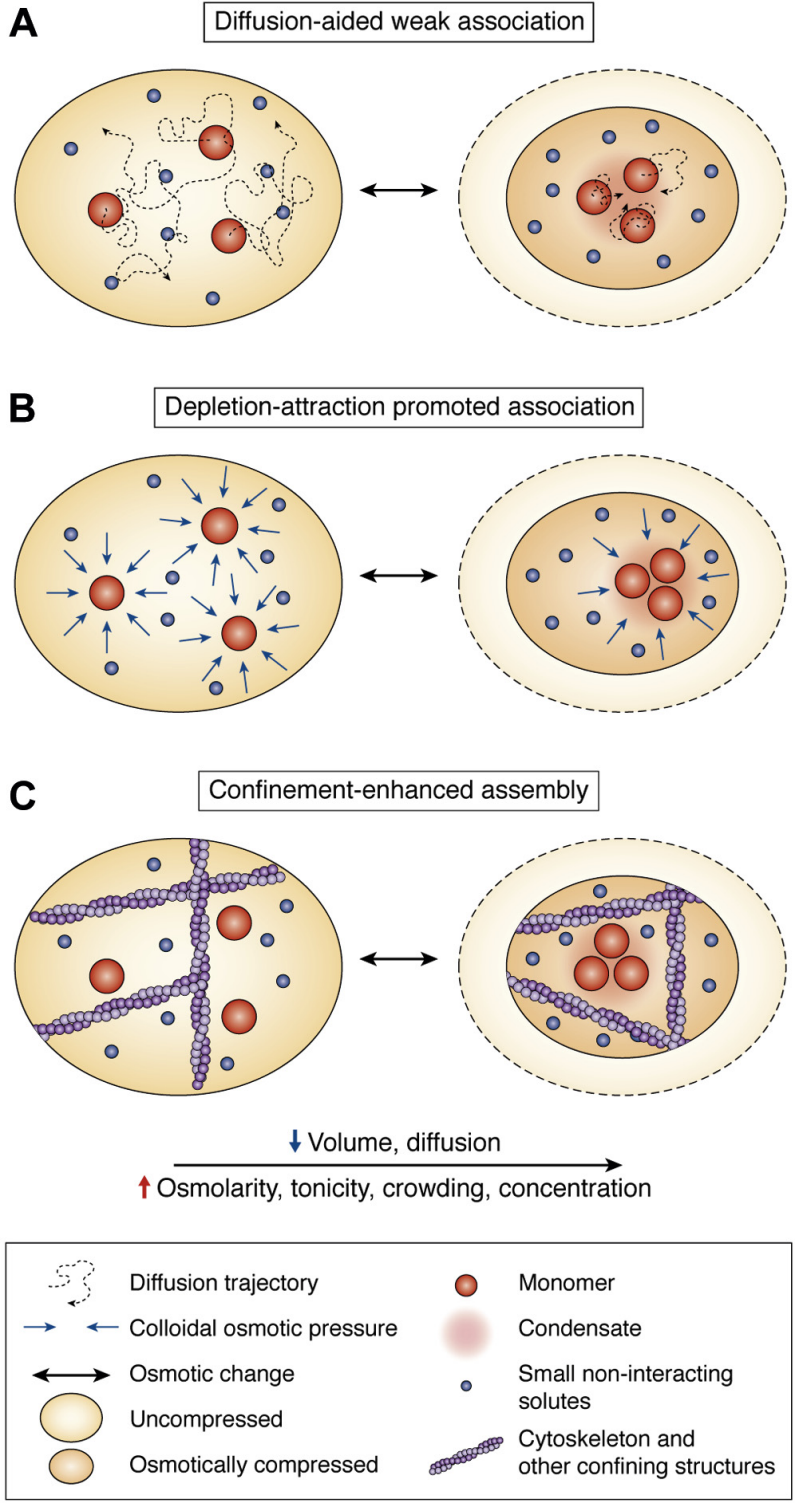
**Consequences of hyperosmotic volume change on phase separation.***A*, increased packing slows down intracellular diffusion, favoring associations mediated by weak interactions. *B*, depletion attraction maximizes entropy of a system by promoting association of larger solutes. *C*, the presence of compartments between rigid intracellular structures leads to confinement effects that can favor association and phase separation processes.

Proteomes have evolved to maintain a certain degree of molecular crowding inside the cell, where ∼30% of the space is occupied by macromolecules, by selecting for net repulsion among proteins *via* surface negative charges (109). Under optimal conditions, this net repulsion may serve to keep proteins from aggregating. However, an increase in crowding by osmotic compression can perturb this balance and overcome the net repulsion, thereby allowing proteins at the saturation concentrations to demix and undergo condensation (Fig. 1). The increased packing and reduced volume upon osmotic compression also result in a global decrease in molecular diffusion. The diffusion rate generally is an important factor influencing intracellular biochemistry. Diffusion can affect phase separation in multiple ways: the growth of an MLO requires a supply of free building blocks, and this supply can be limited by diffusion; however, weak association reactions may instead be favored by slowed diffusion (Fig. 3*A*). The rate of weak, diffusion-limited association reactions is thus expected to increase with crowding both by the increase in net concentration and by the decreased diffusion of the reactants (110). Experimental evidence for the idea that cells can potentially modulate the degree of crowding or the assembly of MLOs to regulate biochemical processes through diffusion is, however, relatively new. Joyner *et al.* (53) suggested that in yeast, volume reduction during glucose starvation regulates intracellular protein mobility. Delarue *et al.* (111) demonstrated that mTORC1 signaling can tune the extent of molecular crowding in cells by modulating the number of ribosomes, thereby modulating phase separation. It remains to be established what other mechanisms exist that modulate crowding and phase separation and how universal such mechanisms are.

Crowding also influences molecular interactions *via* the excluded volume effect (112). This effect, also called depletion attraction, predicts that in crowded solutions containing solutes of different sizes, the aggregation of the large solute can increase the entropy of the system, and the colloidal osmotic pressure of the smaller solute upon the larger particle can prevent dissociation of complexes. Under these conditions, the theory of depletion attraction predicts that association of large macromolecules can effectively increase the entropy of the system, thus promoting phase separation processes (Fig. 3, *A*–*B*). Recent evidence points to the possibility that cells utilize mechanisms that modulate molecular crowding by regulating the density of both small and large solutes (111). Among other methods, genetically encoded nanoparticles (GEMs) are emerging as powerful tools for measuring the underlying crowding and rheology *via* intracellular single-particle tracking (111, 113, 114).

Rigid cytoskeletal elements such as actin and microtubule fibers occupy about 20% of the cell so that cytosolic macromolecules are thought to reside, on average, only a couple of molecular radii from at least one cytoskeletal element (115). This has led to the notion that in addition to being in a crowded environment, cytosolic proteins exist in a state of “confinement,” where their diffusion is highly restricted and limited by such structures. Theoretical work has demonstrated how changes in confinement and in molecular crowding can influence both folding and aggregation of proteins, indicating that changes in intracellular confinement may be another factor that promotes condensation in response to osmotic compression (116, 117). Depending on the nature of confinement, molecular association is expected to give rise to structures such as globular aggregates and long, rod-like structures (Fig. 3*C*). Indeed, cellular proteins have been observed to form such structures, although the mechanisms that drive their formation remain to be established. Various observations of dynamic higher-order organization of metabolic enzymes in eukaryotes have been reviewed by O’Connell *et al.* (118). Similarly, Webb *et al.* (119) observed that phosphofructokinase undergoes redistribution to foci and filaments upon challenge with citrate. These findings illustrate the types of higher-order structures that may arise under physiological and stress conditions, providing evidence for a widespread and ubiquitous role of crowding and confinement in organizing and assembling MLOs.

## Clouds in the cell: reconceptualizing intracellular organization

The term “condensate” has been used in the literature alongside some everyday metaphors for liquid–liquid phase separation, such as the formation of immiscible droplets in vinaigrette or lava lamps (2). These examples capture the thermodynamics of demixing, where the energy of the vinaigrette system is minimized when oil and vinegar undergo phase separation. However, this analogy is limited because it suggests that the two components of the mixture exist in stable, mutually exclusive phases. In biological contexts, phase separation more typically leads to an enrichment of components in one or the other phase, and the degree of partitioning is relevant to understanding the gain or loss of activity in the more concentrated phase. Furthermore, while LLPS appears to be widespread, maturation of liquid-like droplets into gel- and solid-like states is a pervasive phenomenon not captured by the oil–water analogy. As the study of MLOs in physiological and disease contexts becomes more widespread, an additional analogy may be beneficial to serve as a model for biologists.

The study of phase separation has extensively used cloud-related terminology in the more distant past. In the study of protein precipitation, for example, the temperature at which a protein solution turns opaque due to phase separation of the protein is denoted as T_cloud,_ or the cloud point, above which the solution is constituted of a single phase (120). The cloud point therefore represents the optimal conditions of concentration and other physicochemical factors that allow a protein to traverse the phase boundary from a vapor-like state to a condensed state (Fig. 1). Here we reintroduce the analogy of cloud formation that has previously been alluded to in the context of biological LLPS (16, 20, 121, 122). This metaphor emphasizes the rapid, highly reversible transition from a dispersed to a more condensed phase characteristic of phase separation responses to stress.

The cloud-formation metaphor takes us beyond merely the assembly of droplets. It intuitively allows us to make specific predictions related to the impact of physical variables such as temperature (kinetic motion) and “humidity” (relative component concentration) to condensation. It also renders intuitive predictions about possibilities for intracellular condensates that are not currently reported, such as the potential for “smog,” where a condensate of one type is nucleated or otherwise influenced by components that do not otherwise constitute it. It provides a rich language to describe condensates based on a continuum of physical properties—“vapor/mist” *versus* “droplets” *versus* frozen/hardened “hail.” Finally, it provides a new conceptual model of mesoscale organization biology that draws from a physical system that is intrinsically emergent and possesses fractal properties.

Consider the highly studied case of TDP43 fibrillization in ALS. TDP-43 under physiological conditions has been found to condense into dynamic, liquid-like droplets in the nucleus and shows condensation behavior in the cytosol upon exposure to preexisting TDP-43 fibrils. Cytosolic TDP-43 droplets formed upon deletion of the protein’s nuclear localization signal were found to mature into less dynamic, gel-like structures upon arsenite stress (122, 123). Similarly, FUS protein, also associated with ALS pathologies, condenses under normal conditions, but these condensates show liquid-to-solid transitions, and this tendency is enhanced by disease mutations (124, 125). These examples highlight how vapor–liquid–solid transitions may represent a universal, intrinsic tendency of multivalent biopolymers under physiological conditions. The resulting condensates can undergo maturation/solidification upon exposure to specific environmental or biochemical perturbations, resulting in both altered material properties of the condensates and consequences for cellular homeostasis, including pathologies.

The emerging picture is therefore one of a pervasive potential for multivalent molecules to be either within the two-phase regime or poised on the phase boundary between a “vapor”-like dispersed state and a more condensed phase (Fig. 3). This allows such molecules to rapidly transition to more condensed phases in response to intrinsic- and extrinsic perturbations, albeit in a highly regulated and carefully tuned manner. This hypothesis is consistent with the existence of dedicated cellular mechanisms that serve to promote the dissolution of condensates (1, 46, 49). Moreover, the resulting condensates can protect the cell by suspending vital cellular functions until the perturbations cease, but alternatively are then susceptible to pathogenic maturation into gel-like or solid states that can result in toxicity.

In conclusion, as our insights into intracellular organization by phase separation expand, laying the foundation for understanding how phase separation pervasively regulates cellular function and survival, we also learn about the selection pressures that shape our proteome. It is our hope that the additional metaphor proposed here of phase separation as a form of intracellular “cloud formation” may facilitate the intuition needed to appreciate the associated range of phenomena as readily reversible, highly adaptive cell reorganization responses to internal and external cues.

## Conflicts of interest

The authors declare that they have no conflicts of interest with the contents of this article.
